# Indigestion in Children

**Published:** 1886-03

**Authors:** Wm. H. Hale


### Indigestion in Children.

BY WM. H. HALE, M.D.

Children are particularly prone to disorders of the digestive apparatus, and the most prevalent form is indigestion.

During the summer especially, owing to the extreme heat and the ingestion of food entirely unsuited to the age of the child, is this the case.

Very frequently, on questioning the mother as to whether she had given anything but the breast to an infant of a few months, I have been informed that she gave it a taste of meat, potato, or some other article which it is unable to assimilate. The gastric and intestinal secretions are not of sufficient quality or quantity to effect their digestion, consequently they lie in the stomach acting as an irritant, and ultimately vomiting takes place. Previous to the occurrence of vomiting, fermentation develops, and the injected matter is of an extremely acid character. It seems entirely beyond her comprehension that she is giving a poison to the child, and is slowly starving it, from the fact that the absorbents are unable to take it up to supply the wants of the economy.

On first seeing a child with an attack of indigestion, one may be puzzled as to what the affection is. We generally find the child, who but a few hours previous was bright and playful, now listless, languid, lying down, with no interest in his playmates, eyes bright and feverish looking, skin hot, and temperature high, rising in a few hours to 100° F., and even 103° F., or 104° F. The fever generally continues until the removal of the irritating matter from the stomach, when rapid defervescence occurs. Sometimes there are small diarrhœal stools, but more frequently constipation exists. If the child is old enough to express itself, it will complain of headache, excessive thirst, and nausea. Vomiting generally occurs.

Extreme prostration occurs, and the patient becomes limp, from the severity of the symptoms. Coldness and chilliness are at times complained of, but this is only transient, and is soon succeeded by extreme heat. Vomiting is at times severe, and large amounts of food are expelled from the overloaded stomach, in the same condition as when it was eaten; accompanying it is some mucus, and at times bile. After the expulsion of these matters and a free evacuation of the bowels, the symptoms quickly subside, and a return to health takes place.

The causes of this affection are numerous, but I will only mention a few which are especially prevalent during the hot season.

The ingestion of large amounts of fruit or vegetables, and particularly corn, no doubt from its known indigestible qualities, and large draughts of ice water, which chill the stomach, preventing digestion, are the most frequent causes, and the ones which have come most frequently under my notice. At times we find it due to too frequent nursing, the child being given the breast every time it cries, if that is every five minutes during the day. Children should be nursed at an interval of two hours for the first few months, and as they get older it should be increased in length.

The treatment is extremely simple, so soon as we are positive that it is indigestion. I find a calomel purge, together with a fever mixture, generally all that is necessary. Quinine is one of the best fever depressants, as it also combines tonic properties. The regulation of diet, together with the above measures, and subsequently a tonic, is all that is necessary to complete recovery.

